# Stopped Flow of Glycerol Induces the Enhancement of Adsorption and Aggregation of HRP on Mica

**DOI:** 10.3390/mi14051024

**Published:** 2023-05-10

**Authors:** Yuri D. Ivanov, Ivan D. Shumov, Andrey F. Kozlov, Maria O. Ershova, Anastasia A. Valueva, Irina A. Ivanova, Vadim Y. Tatur, Andrei A. Lukyanitsa, Nina D. Ivanova, Vadim S. Ziborov

**Affiliations:** 1 Institute of Biomedical Chemistry, Pogodinskaya Street, 10 Build. 8, Moscow 119121, Russia; shum230988@mail.ru (I.D.S.); afkozlow@mail.ru (A.F.K.); motya00121997@mail.ru (M.O.E.); varuevavarueva@gmail.com (A.A.V.); i.a.ivanova@bk.ru (I.A.I.); ziborov.vs@yandex.ru (V.S.Z.); 2 Joint Institute for High Temperatures of the Russian Academy of Sciences, Moscow 125412, Russia; 3 Foundation of Perspective Technologies and Novations, Moscow 115682, Russia; v_tatur@mail.ru (V.Y.T.); andrei_luk@mail.ru (A.A.L.); ninaivan1972@gmail.com (N.D.I.); 4 Faculty of Computational Mathematics and Cybernetics, Moscow State University, Moscow 119991, Russia; 5 Moscow State Academy of Veterinary Medicine and Biotechnology Named after Skryabin, Moscow 109472, Russia

**Keywords:** glycerol flow, enzyme adsorption, horseradish peroxidase, atomic force microscopy

## Abstract

Glycerol is a usable component of heat-transfer fluids, and is thus suitable for the use in microchannel-based heat exchangers in biosensors and microelectronic devices. The flow of a fluid can lead to the generation of electromagnetic fields, which can affect enzymes. Herein, by means of atomic force microscopy (AFM) and spectrophotometry, a long-term effect of stopped flow of glycerol through a coiled heat exchanger on horseradish peroxidase (HRP) has been revealed. Samples of buffered HRP solution were incubated near either the inlet or the outlet sections of the heat exchanger after stopping the flow. It has been found that both the enzyme aggregation state and the number of mica-adsorbed HRP particles increase after such an incubation for 40 min. Moreover, the enzymatic activity of the enzyme incubated near the inlet section has been found to increase in comparison with that of the control sample, while the activity of the enzyme incubated near the outlet section remained unaffected. Our results can find application in the development of biosensors and bioreactors, in which flow-based heat exchangers are employed.

## 1. Introduction

Glycerol is a trihydric alcohol, which is widely used in industry [1,2]. Pauling noted the usability of glycerol as a component of antifreezes [1]; in this regard, glycerol represents a safe alternative to ethylene glycol, which is known to be toxic for animals and humans [3]. With regard to its application in biomedical research, the use of glycerol for modulation of the viscosity of solutions to be analyzed [4,5], and for preliminary treatment of surface of biosensor chips [6] was reported. Moreover, Yang et al. emphasized good suitability of glycerol for its use as a component of heat transfer fluids in microchannel-based miniaturized heat exchangers, which are of use in biotechnology and microelectronics [2]. In this regard, it should be emphasized that modern nanotechnology-based biosensors comprise microelectronic components [7,8,9]. Microelectronic chips are characterized by high heat flux densities [10]. Accordingly, providing efficient heat transfer becomes a crucial task of the development of novel biosensor systems. In this connection, apart from traditional heat transfer fluids, one should also mention the so-called nanofluids [11,12], which consist of conventional heat transfer fluids with added metallic or oxide nanoparticles [11]. Akkurt et al. reported that the use of nanofluids allows one to additionally increase the efficiency of heat transfer rate [11].

The above-mentioned biosensors [4,6] and heat exchangers [2] pertain to flow-based systems, in which a flow of glycerol-containing working fluids is organized. In this connection, one should emphasize the possible occurrence of the so-called triboelectric effect, which consists in the generation of electric charge upon the flow of liquid media along solid surfaces [13]. Such an effect was reported for both aqueous [14,15,16,17,18,19] and non-aqueous fluids [20,21,22], including glycerol [23,24]. The triboelectric generation of charge results in an occurrence of electromagnetic fields [18,23,24]. Electric fields and electromagnetic waves can influence biological macromolecules [25]. This circumstance is particularly important in flow-based systems intended for operation with biological macromolecules, such as the above-mentioned biosensors and bioreactor heat exchangers.

Electric [26,27,28,29,30,31], magnetic [32,33,34,35,36] and electromagnetic [37,38,39,40,41,42,43,44,45,46,47] fields are known to affect enzymes. In many papers, the effects of pulsed electric [26,27,28,29,30,31] and electromagnetic [42] fields on enzymes are considered. It is to be emphasized that the effect of pulsed electric field (PEF) can be quite different, and depends on the enzyme type [27,31] and treatment conditions [26,27]. On the one hand, high-voltage (approximately 27 to 42 kV/cm) 126-µs-long PEF treatment was reported to lead to a considerable (by tens of %) inactivation of many enzymes, such as pepsin, peroxidase, and polyphenol oxidase [31], and lipase [26], while lysozyme was virtually unaffected by 38 kV/cm PEF after a 126-µs-long treatment [31]. On the other hand, Ohshima et al. [27] revealed an enhancing effect of ≤12 kV/cm PEF on peroxidase and β-galactosidase. These authors observed a 20% increase in the activity of peroxidase and β-galactosidase after exposure of these enzymes to 12 and 13 kV/cm PEF for 30 and 60 s, respectively [27]. At that, these enzymes were found to lose their activity after treatment with stronger PEF [27]. Regarding magnetic fields, Wasak et al. [32] demonstrated that the action of a low-frequency rotating magnetic field can either enhance or suppress the enzymatic activity of horseradish peroxidase (HRP) against o-dianisidine depending on the solution pH, field parameters, and exposure time. Earlier, by atomic force microscopy (AFM), Sun et al. [35] found that HRP forms extended structures on mica after exposure to alternating magnetic field, while the enzymatic activity was hardly affected. In this way, these authors demonstrated the advantages of using AFM for studying the interaction of applied magnetic fields with an enzyme [35,36].

Similar to the case with PEFs, the effect of electromagnetic fields (EMFs) on the activity of various enzymes was also reported to be either positive (enhancing) or negative (suppressing) [41,45,46] depending on the EMF frequency and the enzyme type. Morelli et al. observed that membrane-associated enzymes (phopsphoglycerate kinase, alkaline phosphatase, adenylate kinase) lose their activity after the exposure to 75 Hz EMF, while other enzymes (for instance, succinic dehydrogenase) were found to be insensitive to such an EMF [45]. In contrast, Thumm et al. revealed a stimulation of cAMP-dependent protein kinase by a very low-frequency (20 Hz) EMF [46]. HRP was found to lose its catalytic activity after the exposure to a 50 Hz, 2.7 mT EMF, while being insensitive to the EMF with 2-times higher (100 Hz; 5.5 mT) frequency [41]. Regarding EMFs of higher frequencies (radiofrequency EMFs, RF EMFs), interesting results were reported for HRP enzyme. Fortune et al. did not reveal any non-thermal effect of RF EMFs of various frequencies (13.56 MHz, 915 MHz, and 2.45 GHz) on HRP [38]. Yao et al. observed a slight activation of HRP upon 27.12 MHz RF heating at high power (6 kW) and 50 °C temperature, while treatment at the same frequency but at higher temperatures resulted in the loss of enzymatic activity of HRP [44]. Latorre et al. observed a dramatic inactivation of peroxidase from red beet and polyphenoloxidase by microwave treatment [39]. 

The data listed above clearly indicate that the effect of electric and electromagnetic fields enzymes can vary, and further investigation is required in order to better understand the phenomenon of the interaction of external fields with enzymes. Here, we must emphasize that in the majority of studies listed above, macroscopic methods in solution (such as spectrophotometry) were employed for the determination of changes in the properties of enzymes. In the macroscopic methods, the signal from a large ensemble of the molecules under study is acquired. This leads to the fact that subtle effects of external fields on enzyme macromolecules can well remain unrevealed by macroscopic methods [35,36,48]. In this regard, high-resolution methods are of use, and AFM is one of them. In a number of papers, it was demonstrated that AFM allows one to investigate changes in physicochemical properties of enzymes at the level of single molecules [35,36,49,50], thus revealing even subtle effects (which are often indistinguishable by macroscopic methods [35,36,48]) of external fields on the enzymes under study, including the effects caused by action of weak EMFs [47]. 

Herein, we demonstrate how our well-established approach, which consists in a combined use of AFM-based adsorption studies and spectrophotometry-based estimation of enzymatic activity, has allowed us to reveal a long-term effect of glycerol flow in a heat exchanger on the properties of HRP, which has been used as a model enzyme, as was proposed in a number of published papers [41,44,47,48]. Previously, we demonstrated that the electromagnetic field induced by the flow of glycerol can affect both the adsorption properties and the enzymatic activity of HRP [24]. That is, the effect of glycerol flow is so strong that the changes in the enzyme properties can be revealed by the macroscopic method (spectrophotometry)—as opposed to the subtle effects [48]. Our present study is aimed at further investigating the influence of glycerol flow on enzymes with the example of HRP. In this connection, the first question is whether the electromagnetic field, induced by glycerol flow, can have a long-term effect on the enzyme. This has not yet been studied. Another question is whether this effect will be evident at the macroscopic level. With this purpose, herein, the glycerol flow through the heat exchanger had been stopped prior to the incubation of HRP in the experimental setup. Only after stopping the flow was the HRP solution incubated near the linear inlet and outlet sections of the heat exchanger. This is the major difference with our experiments reported previously [24], when glycerol was pumped continuously without stopping its flow before the experiment. In this previous paper, electromagnetic fields, generated by the flow of glycerol through the linear sections of a heat exchanger (coiled polymeric pipe), were found to induce an increased aggregation of HRP enzyme upon its adsorption onto mica. Herein, for the first time, we have revealed that there is a long-term effect on an enzyme incubated near these sections of the heat exchanger after stopping the glycerol flow. Our recent results reported herein should thus be taken into consideration in the development of biosensors and bioreactors containing flow-based heat exchangers, in which non-aqueous heat-transfer fluids are utilized.

## 2. Materials and Methods

### 2.1. Chemicals and Protein

HRP enzyme (peroxidase from horseradish; Cat. #6782) and its substrate 2,2′-azino-bis(3-ethylbenzothiazoline-6-sulfonate) (ABTS) were purchased from Sigma. Disodium hydrogen orthophosphate, citric acid, and hydrogen peroxide were purchased from Reakhim (Moscow, Russia). 2 mM Dulbecco’s modified phosphate buffered saline (PBSD buffer) was prepared from a salt mixture purchased from Pierce (Waltham, MA, USA). All solutions were prepared using deionized ultrapure water (with 18.2 MΩ  · cm resistivity) obtained with a Simplicity UV system (Millipore, Molsheim, France). Glycerol was purchased from Glaconchemie GmbH (Merseburg, Germany).

### 2.2. Experimental Setup

**Figure 1:**
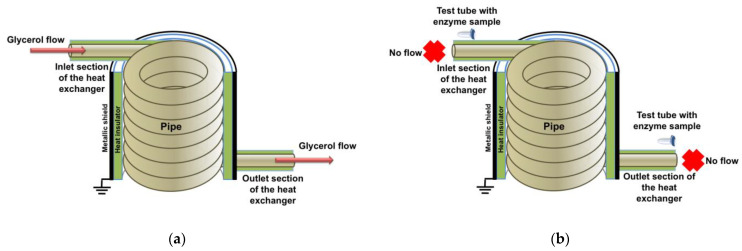
The setup before (**a**) and at the moment of (**b**) starting the incubation of HRP samples.

Figure 1 schematically shows the experimental setup. In general, this setup is analogous to that described in detail in our previous paper [24]. 

In brief, the heat exchanger was modeled by a polymeric coiled pipe, through which glycerol was pumped. The latter was warmed up to 65 °C in order to provide its fluidity, making it possible to pump at 9 L/s flowrate. The coiled section of the heat exchanger was ground-shielded in order to avoid any interference from the electromagnetic field generated in it. Moreover, the entire pipe (both the coiled and the linear sections) were heat-insulated in order to avoid heating of the enzyme samples, which can affect the enzyme properties [44]. The temperature of the enzyme samples was equal to the room one (25 °C).

Prior to the experiment, the glycerol flow had been pumped through the heat exchanger for 40 min, and then the flow was completely stopped. After stopping the flow, 1-mL samples of the enzyme solution under study (0.1 µM HRP in 2 mM PBSD, pH 7.4) were placed 20 cm away from the ground-shielded coil near either the inlet or the outlet section of the heat exchanger, so that the distance between the pipe and the test tube with the sample was 1 cm. The samples were incubated near the experimental setup for 40 min (as in our previously reported experiments [24]). The control enzyme samples were incubated three meters away from the experimental setup.

### 2.3. AFM Measurements

HRP samples for AFM measurements were prepared by direct surface adsorption [51] according to the well-established technique employed in our previous experiments [24]; this technique is described in detail elsewhere [18,23,24,47,48]. The AFM scanning was performed with a Titanium AFM (NT-MDT, Zelenograd, Russia; the microscope pertains to the equipment of “Human Proteome” Core Facility of the Institute of Biomedical Chemistry, supported by Ministry of Education and Science of Russian Federation, agreement 14.621.21.0017, unique project ID: RFMEFI62117X0017) using NSG10 cantilevers with tip curvature radius of 10 nm. For each AFM substrate, no less than 16, 2 µm × 2 µm, scans in different substrate areas were obtained.

The total number of objects, visualized on each AFM substrate with adsorbed HRP, was ≥200. Blank experiments were performed with pure PBSD instead of HRP enzyme solution, and no objects with heights exceeding 0.5 nm were revealed in these blank experiments. AFM operation and data treatment was performed with NOVAPx software (NT-MDT, Moscow, Zelenograd, Russia) supplied with the atomic force microscope. The number of the visualized particles in the obtained AFM images was calculated automatically using specialized AFM data processing software developed at the Institute of Biomedical Chemistry.

The distribution of the AFM-visualized objects with heights (density functions), and the number of particles visualized by AFM (normalized per 400 µm^2^) were calculated as described by Pleshakova et al. [52].

### 2.4. Spectrophotometric Measurements

In parallel with the AFM measurements, the enzymatic activity of HRP in the samples incubated as described in Section 2.2, was estimated by spectrophotometry. With this purpose, the well-established technique developed by Sanders et al. [53] has been employed. In brief, the activity of HRP against its substrate ABTS has been estimated at pH 5.0 (as recommended by the enzyme manufacturer [54]) in phosphate-citrate buffer. The use of phosphate-citrate buffer was reported to be optimal for the HRP-ABTS assay [55]. The absorbance of 1 nM HRP solution (containing 0.3 mM ABTS and 2.5 mM H_2_O_2_) at 405 nm was monitored for 5 min, and the resulting dependencies were plotted in the form of absorbance vs. time (*A*_405_*(t)*) curves. The enzymatic activity was estimated based on the first 120 s of observation, and the calculations were performed as described previously [56].

## 3. Results

### 3.1. Atomic Force Microscopy

**Figure 2:**
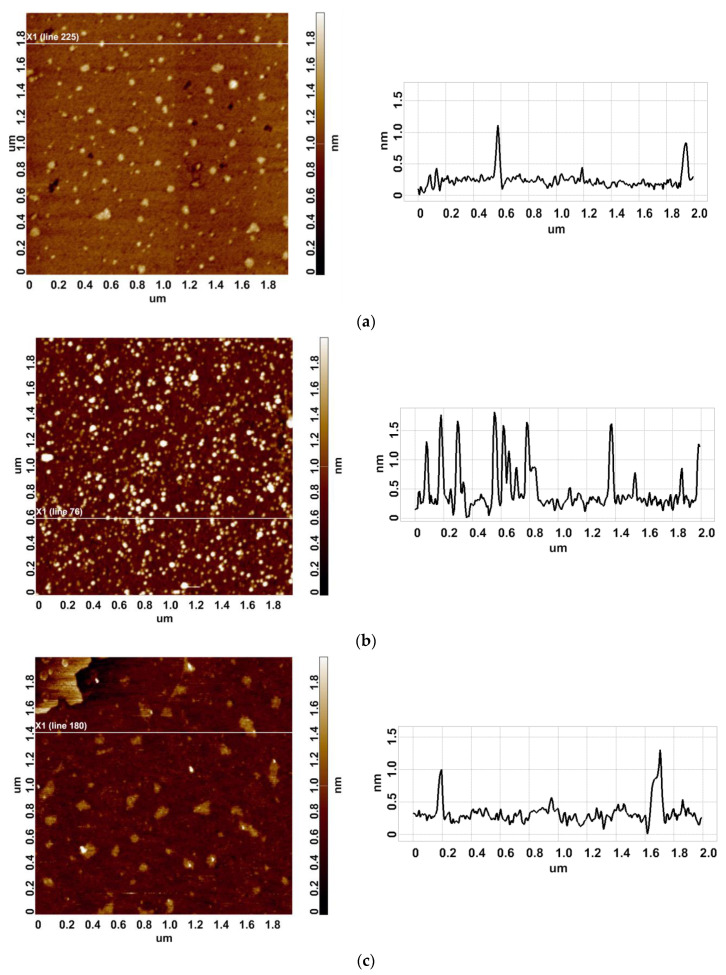
Typical AFM images (panels on the left) and cross-section profiles (panels on the right) of mica-adsorbed HRP obtained in control experiments (in which the HRP solution was incubated 3 m away from the setup; panel (**a**)) and in working experiments (in which the HRP solution was incubated near the setup; panels (**b**,**c**)).

As was noted in the Introduction, we have previously revealed an increased HRP aggregation under the influence of a continuous glycerol flow [24]. In the continuation of this research, we have performed further experiments in order to find out whether the incubation near either the inlet or the outlet section of a coiled heat exchanger after stopping the glycerol flow will affect the adsorption properties of HRP on mica. The heights of HRP adsorbed onto mica from its 0.1 µM solutions, which were incubated either near of far away from the experimental setup with stopped glycerol flow, have been measured by AFM. Figure 2 displays typical AFM images and cross-section profiles of mica-adsorbed HRP obtained in working experiments (in which the HRP solution was incubated near the setup) and in control experiments (in which the HRP solution was incubated 3 m away from the setup).

In the control experiments, 1–1.2-nm-high compact objects were visualized (Figure 2a). These objects have been attributed to HRP particles, since no objects with >0.5 nm was revealed in the blank experiments with pure PBSD. Similar to the control experiments, in the working experiments, compact objects have also been visualized (Figure 2b,c). Accordingly, in order to find out whether there is the difference between the working and the control samples, the density function plots obtained in the experiments should be analyzed. 

**Figure 3:**
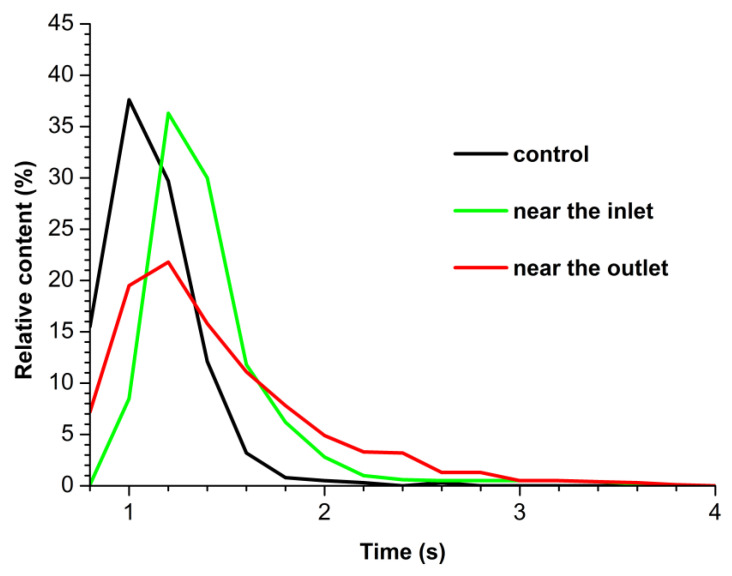
Density function plots obtained for control HRP sample (black curve) and for working HRP samples, when the enzyme solution was incubated either near the inlet (green curve) or near the outlet (red curve) section of the heat exchanger.

Figure 3 displays density function plots obtained for control HRP sample and for working HRP samples (incubated either near the inlet or near the outlet section of the heat exchanger).

The density function *ρ*(*h*) plot obtained for the control HRP sample (Figure 3, black curve) has the maximum at *h_max_* = 1.0 ± 0.2 nm, while its width at half-height (WHH) is 0.5 nm. We have previously justified that in the case of HRP (whose molecular weight makes up 40 kDa [57]) adsorbed onto bare mica, objects with this height should be considered as a monomeric form of this enzyme [24]. 

**Figure 4:**
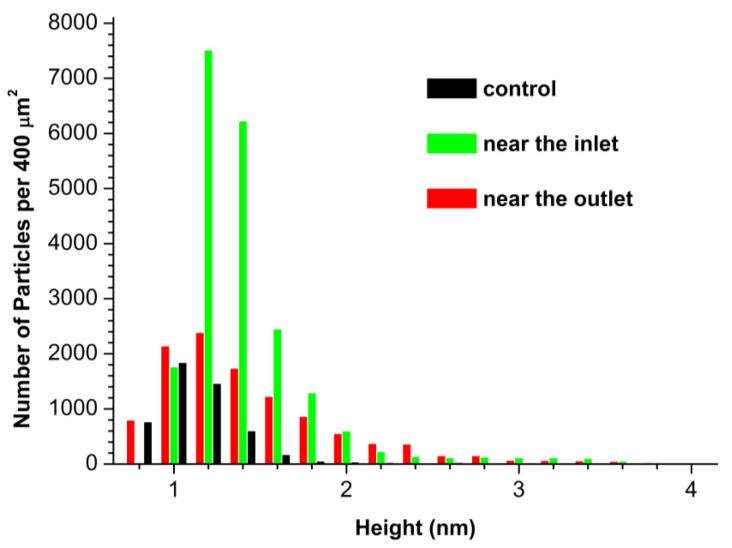
Number of AFM-visualized particles, normalized per 400 µm^2^, obtained for control HRP sample (black bars) and for working HRP samples, when the enzyme solution was incubated either near the inlet (green bars) or near the outlet (red bars) section of the heat exchanger.

In case of the HRP sample incubated in the working experiments near the inlet and the outlet sections of the heat exchanger, the maxima of the resulting *ρ*(*h*) plots shift towards greater heights (*h_max_* ≈ 1.4 nm). It is interesting to emphasize that, while for the sample incubated near the inlet, the WHH is the same as for the control sample (WHH = 0.5 nm), the *ρ*(*h*) plot for the sample incubated near the outlet becomes considerably broader (WHH = 0.8 nm), showing an increased contribution of objects with increased heights to the plot’s right wing. The objects with increased heights can be well attributed to the aggregated HRP. Importantly, for the sample incubated near the outlet, the formation of higher-order HRP aggregates with heights of 2.4 nm is considerable (Figure 3, red curve). For the working and control samples, Figure 4 displays the number of AFM-visualized particles, normalized per 400 µm^2^ area of the substrate surface.

For both working HRP samples, which were incubated either near the inlet (Figure 4, green bar; *N*_400_ = 20 675) or near the outlet (Figure 4, red bar; *N*_400_ = 10 900) section of the heat exchanger, the *N*_400_ value was considerably higher than that obtained for the control HRP sample (black bar; *N*_400_ = 4867). These data indicate an increase in the number of HRP particles adsorbed on mica.

### 3.2. Spectrophotometry

**Figure 5:**
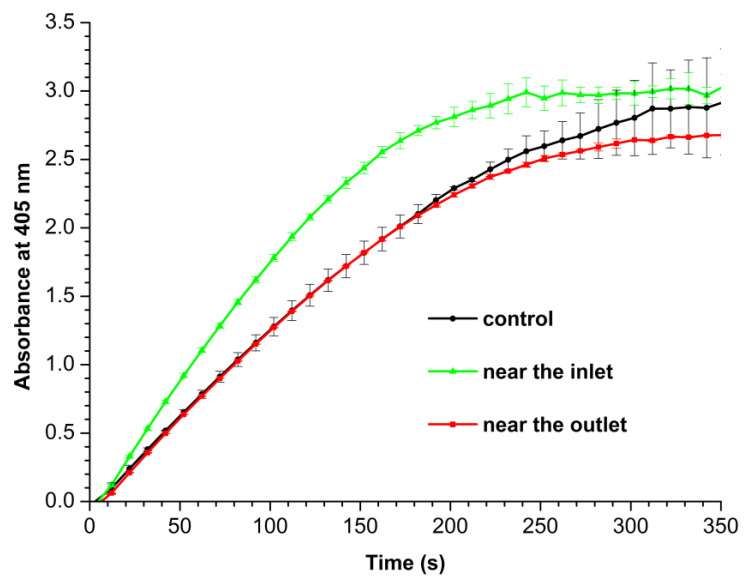
Absorbance at 405 nm vs. time (*A*_405_(*t*)) curves recorded for control HRP sample (black curve), and for working HRP samples, when the enzyme solution was incubated either near the inlet (green bars) or near the outlet (red bars) section of the heat exchanger.

Figure 5 displays *A*_405_(*t*)) curves recorded for working and control enzyme samples.

The curves shown in Figure 5 indicate that the amount of ABTS substrate converted by HRP during the 5-min-long measurement is similar for the control and both working samples. One should emphasize that the shape of the *A*_405_(*t*) curve recorded for the enzyme sample incubated near the inlet section of the heat exchanger is markedly different from those recorded for both the control enzyme sample and the sample incubated near the outlet, indicating higher enzymatic activity of HRP in the sample incubated near the inlet. Based on the first 120 s of observation, the analysis of the slope of the *A*_405_(*t*) curves gives the following values of HRP enzymatic activity: 237.8 U/(mL enzyme) in the sample incubated near the inlet, 200.9 U/(mL enzyme) in the sample incubated near the outlet, and 201.4 U/(mL enzyme) in the control sample. That is, after the incubation near the inlet section, the enzymatic activity of HRP has increased by 19% in comparison with both the sample incubated near the outlet and the control sample. It should be noted that in the case of continuous flow of glycerol, the change in the activity of HRP incubated near the inlet was comparable with the error, and in the case of incubation near the outlet, a 18% decrease in the enzymatic activity was observed [24].

## 4. Discussion

We have investigated the influence of stopped glycerol flow in a coiled heat exchanger on the HRP enzyme, whose solution was incubated near either the inlet or the outlet linear section of the heat exchanger. In our experiments, an increased aggregation of the enzyme on mica has been revealed by AFM. Namely, an increased contribution of AFM images of objects with greater heights has been observed upon analysis of density function plots obtained for HRP samples incubated near the heat exchanger with stopped glycerol flow—in contrast to the control HRP samples. This situation is similar to the one observed for flowing glycerol, which was described in our previous paper [24]. It is important to emphasize that the effect on the enzyme has been observed after stopping the glycerol flow, and after the incubation time was 40 min. Such a long-term effect can be explained by long-lived excitements, which result from the formation of nanobubble clusters, as was noted by Bunkin et al. [58]. These authors observed the formation of nanobubble clusters upon electromagnetic excitation of aqueous solutions by an external electromagnetic field with a fluorescence-based technique. The formation of nanobubbles is discussed in several papers [59,60,61,62]. In our reported experiments, an electromagnetic field is induced upon the flow of a non-aqueous fluid (glycerol) in the polymeric pipe at the expense of triboelectric generation of charge [20,21,22,23,24]. 

Furthermore, it should be emphasized that our enzymatic activity measurements performed by spectrophotometry revealed a 19% increase in the HRP activity in the sample incubated near the inlet—in contrast to both the sample incubated near the outlet and the control sample. This is a very interesting result. Of note, external electric, magnetic and electromagnetic fields were previously shown to be able to enhance the enzymatic activity of HRP under certain experimental conditions. Ohshima et al. reported an increase in the activity of peroxidase after its 30 s exposure to a 12 kV/cm pulsed electric field [27]. Wasak et al. observed 8% and 12% increase in the activity of HRP against o-anisidine at pH 4.5 after treatment of the enzyme in 1 Hz (5.39 mT) and 20 Hz (12.02 mT) rotating magnetic field, respectively [32]. Yao et al. reported that radio frequency heating (27.12 MHz, 6 kW, 50 °C) of HRP leads to a slight (by 5.33 to 13.73%) activation of HRP; these authors performed their measurements at pH 6.1 with the use of guaiacol as HRP substrate [44]. In these studies, a suppression of the enzymatic activity of HRP was observed under conditions different from the ones specified above. Herein, we have demonstrated the enhancement of HRP activity as a result of long-term effect of glycerol flow on the enzyme.

Enzyme aggregation often leads to a deactivation of enzymes [63]. Nevertheless, Sun et al. demonstrated that the action of alternating magnetic field on HRP induces changes in its adsorption behavior on mica, leading to the formation of extended quasi-one-dimensional assemblies of the enzyme macromolecules on the substrate surface, as was observed by AFM [35]. At that, the enzymatic activity of HRP against 3,3,5,5-tetramethylbenzidene was hardly suppressed (by not more than 15%). In their earlier paper, these authors showed that external magnetic fields can have a stimulating effect on surface-adsorbed HRP [36]. This can be explained by the difference in the interactions of the enzyme macromolecules with each other and with the solvent, which take place in the presence and in the absence of the mica substrate. That is, we have observed increased aggregation of HRP on the surface of mica substrates, but the spectrophotometry measurements have been performed in solution, where there was no interaction of the enzyme macromolecules with the substrate surface. The phenomena observed in our experiments can be explained by the long-term effect of the stopped flow of glycerol on the hydration shells of the enzyme macromolecules. Indeed, the structure of hydration shell was shown to be one of the factors influencing the functioning of enzymes [64]. Their enzymatic activity can be affected by alterations in their hydration shells [64,65,66]. This is what we have observed in the case with HRP incubated near the inlet section of the heat exchanger. The alterations in the HRP hydration shell have also led to an enhancement of the adsorption and aggregation of the enzyme on mica. This is how we explain the effect of glycerol flow on HRP enzyme in our particular case.

It was shown that shape of objects and moving media can influence the properties of biological molecules. This effect was called Ivanov-Tatur memory effect [67]. Namely, Shipov [67] emphasized the effect of stopped medium on the properties of biological system, which had been incubated near an outlet section of a coiled system. In our present study, we have demonstrated how the stopped flow of glycerol influences HRP upon its incubation near the either inlet or the outlet sections of a coil. The key point is the registration of the effect when initiation of the excitation of the medium is finished, but the medium is still in excited state. The characteristic time of this state is determined by the relaxation properties of the medium [67].

## 5. Conclusions

It is to be emphasized that in this study we have observed the long-term effect on the HRP enzyme, whose incubation near the experimental setup started just after the fluid flow itself had been already stopped—in contrast to the case reported previously [24]. This effect consists in the increased adsorption and aggregation of HRP upon its adsorption onto mica—in comparison with the control enzyme sample. Notably, for the sample incubated near the outlet section of the heat exchanger, the formation of higher-order HRP aggregates with heights of 2.4 nm is more pronounced—in contrast to the sample incubated near the inlet section. Moreover, by spectrophotometry, an enhancement in the enzymatic activity of HRP against ABTS has been revealed in the sample incubated near the inlet section of the heat exchanger—in comparison with both the sample incubated near the outlet and the control sample (in which the enzymatic activities were virtually equal). The phenomena observed can be explained by alterations in the enzyme hydration shell after the exposure to the stopped flow of glycerol [64,65,66]. These alteration lead to a considerable change in the enzyme’s adsorption properties, while its activity was either enhanced or unaffected depending on the position of the sample in the experimental setup. Accordingly, our present research represents one more step towards understanding the effect of how the flow of non-aqueous fluids in heat exchanging units can influence enzyme biomolecules. Heat transfer fluids, employed in heat exchangers, can have various compositions, comprising additional components with different electric and magnetic properties [11]. Accordingly, the possible effects of flows of such complex fluids on biological macromolecules should be thoroughly studied if these fluids are intended for use in processes involving enzymes.

## Data Availability

Correspondence and requests for materials should be addressed to Y.D.I.
